# A new deep-water scavenger species in the genus *Caeconyx* (Crustacea, Amphipoda, Lysianassoidea, Uristidae) from the Porcupine Abyssal Plain

**DOI:** 10.3897/zookeys.1282.191061

**Published:** 2026-06-16

**Authors:** Ben Walker, Michael H. Thurston, Guadalupe Bribiesca-Contreras, Tammy Horton

**Affiliations:** 1 School of Ocean and Earth Sciences, University of Southampton, Southampton, SO14 3ZH, UK National Oceanography Centre Southampton United Kingdom; 2 National Oceanography Centre, Southampton, SO14 3ZH, UK School of Ocean and Earth Sciences, University of Southampton Southampton United Kingdom

**Keywords:** Abyss, amphipods, Atlantic Ocean, deep-sea

## Abstract

*Caeconyx papso***sp. nov**. within the family Uristidae is described from the Porcupine Abyssal Plain, Northeast Atlantic Ocean. The new species differs from the only other member of the genus, *Caeconyx caeculus*, in possessing a triangular, sub-acute eye lobe, a convex and broadly rounded posterodistal margin of epimeron 1, and propodi of pereopods 3 and 4 that are not slender or elongate. Molecular sequence data for COI, H3, 16S, and 28S genes are provided for *C. papso***sp. nov**. Phylogenetic analyses were conducted using this sequence data to assess the relationships between the new species, other members of the family Uristidae, and morphologically allied taxa within the family Tryphosidae. The new species is fully illustrated, and a key to species within *Caeconyx* is presented.

## Introduction

The genus *Caeconyx* Barnard & Karaman, 1991 contains a single species to date, *Caeconyx caeculus* (G.O. Sars, 1891). The species was first described in the genus *Hoplonyx* G.O. Sars, 1891 based on two specimens collected in Lexviken, Trondhjemfjord, Norway at a depth of approximately 275 m (150 fathoms). *Hoplonyx* was later recognised as a junior homonym of the coleopteran genus *Hoplonyx* Thomson, 1858. Stebbing (1906) introduced *Tmetonyx* as a replacement name, and the species formerly assigned to *Hoplonyx*, including *H. caeculus*, were consequently placed in *Tmetonyx*Stebbing 1906. Ruffo (1985) reported the species at bathyal depths in the Mediterranean and transferred the species to *Tryphosella* Bonnier, 1893 on the basis of the short article 3 of gnathopod 1.

Barnard and Karaman (1991: 473) later reassigned *Tryphosella caecula* to the newly erected genus *Caeconyx* Barnard & Karaman, 1991, stating that *Caeconyx* differed from *Tmetonyx* “in the sharp ocular lobe, the short article 3 of gnathopod 1, and the equally extending rami of uropod 3”. When erected, *Caeconyx* was placed in the family Lysianassidae Dana, 1849. The genus has since been moved to the family Uristidae Hurley, 1963, based on the distinct 7/4 crown arrangement of the spine teeth of the outer plate of maxilla 1 (Lowry and Kilgallen 2014).

According to Lowry and Kilgallen (2014), *Caeconyx* differs from the closely related uristid genus *Anonyx* Krøyer, 1838 in having a distally attached mandibular palp, rather than a midway attachment, and the margins of the gnathopod 1 propodus, which are tapering in *Anonyx* but are subparallel in *Caeconyx* (Lowry and Kilgallen 2014).

The Porcupine Abyssal Plain Sustained Observatory (PAP-SO), located in the Northeast Atlantic (48°50'N, 16°30'W) at a water depth of 4850 m, is a key time-series site for the long-term study of the deep ocean (Hartman et al. 2021). The scavenging amphipod time-series at PAP-SO, initiated in 1985, is the longest known abyssal biological time-series in the world and has been vital in understanding the links between scavenging amphipod assemblages, surface ocean processes, and climate (Horton et al. 2020a). Specimens from the PAP-SO study site have also advanced amphipod taxonomy, with material collected for the PAP-SO amphipod time-series contributing to descriptions of four lysianassoid species: *Cyclocaris lowryi* Horton & Thurston, 2014, *Paracallisoma idioxenos* Horton & Thurston, 2015, *Haptocallisoma lemarete* Horton & Thurston, 2015, and *Abyssorchomene patriciae* Hendrycks & De Broyer, 2022. In addition, material collected from the Porcupine Abyssal Plain during ROV surveys has been used to describe the amathillopsid *Amathillopsis inkenae* Lörz & Horton, 2021, and molecular taxonomic work at PAP-SO has revealed an undescribed species of *Eurythenes* S.I. Smith in Scudder, 1882 (Horton et al. 2020b).

Prior to the formal description presented here, in publications on the PAP-SO scavenging amphipod time series (Horton et al. 2020a), *Caeconyx papso* sp. nov. was provisionally identified and assigned to the genus *Paracentromedon* Chevreux & Fage, 1925 with the type-2 temporary taxonomic designation *Paracentromedon* sp. DISCOLL_PAP_52216, according to Horton et al. (2021, 2025). This provisional attribution to the tryphosid genus *Paracentromedon* was revised to *Caeconyx* following comprehensive species dissection and morphological analysis, which revealed a 7/4 crown arrangement of spine teeth on the outer plate of maxilla 1. Mouthpart characters, including the setal-tooth arrangement on the outer plate of maxilla 1, were originally used to separate the two families Uristidae and Tryphosidae Lowry & Stoddart, 1997, with outer plate spine teeth forming a 7/4 crown in Uristidae and a 6/5 arrangement in Tryphosidae (Lowry and Kilgallen 2014). While we attribute *Caeconyx* sp. nov. to the family Uristidae for the purposes of this description, we recognise that numerous independent phylogenetic analyses have indicated the paraphyly of both Tryphosidae and Uristidae within the parvorder Lysianassidira Dana, 1849 (Corrigan et al. 2014; Stewart et al. 2024). A comprehensive phylogenetic analysis of the taxa within the parvorder using both morphological and molecular characters is therefore needed to resolve questions of paraphyly and until this is completed, we include in our analyses those taxa within both families that are closely related to *Caeconyx* morphologically.

Here we describe a new species of *Caeconyx* from the Porcupine Abyssal Plain, extending the known geographic range of the genus from coastal waters of the Northeast Atlantic (Iceland to Trondheimsfjord) and the Mediterranean to the Northeast Atlantic abyssal plain. The discovery of this new species expands the bathymetric range of *Caeconyx* from 150–1095 m to abyssal depths of approximately 4850 m. The new species is illustrated and a key to species of *Caeconyx* is given. We also provide molecular barcode sequences for the new species, and explore the phylogenetic relationships between the new species, other members of the Uristidae, and morphologically allied taxa within the Tryphosidae.

## Materials and methods

Material for the present study was sampled from the Porcupine Abyssal Plain Sustained Observatory (PAP-SO). The material was collected using baited amphipod traps over 11 expeditions to PAP-SO. For details of gear types and sample processing see the relevant cruise reports and Horton et al. (2020a). Details of station numbers, including cruise number and gear type, are given for each specimen in Table 1.

**Table 1.** Station data for the material examined in this study.

Vessel	Cruise	Station	Gear	Latitude (N), longitude (W)	Depth (m)	Date
RRS Challenger	CH6A/85	52216#5	MAR	48.8337°N, 16.5070°W	4842	06/23/1985
RRS Challenger	Ch111	53201#25	DEMAR	48.8183°N, 16.5167°W	4844	04/14/1994
RRS Discovery	D226	13077#04	Ptraps	48.9303°N, 16.5875°W	4844	03/14/1997
RRS Discovery	D226	13077#35	Ptraps	48.9683°N, 16.4155°W	4845	03/19/1997
RRS Discovery	D226	13077#92	DEMAR	48.8250°N, 16.3495°W	4844	03/27/1997
RRS Discovery	D226	13078#3	DEMAR	48.7832°N, 16.3682°W	4842	03/29/1997
RRS James Cook	JC062	JC062#103	ATRAP	49.0970°N, 16.3123°W	4677	08/13/2011
RRS James Cook	JC071	JC071#34	ATRAP	48.9642°N, 16.5020°W	4846	05/05/2012
RRS Discovery	DY077	DY077#61	ATRAP	49.0071°N, 16.3970°W	4846	04/23/2017
RRS Discovery	DY077	DY077#83	ATRAP	49.0065°N, 16.3978°W	4846	04/25/2017
RRS James Cook	JC165	JC165#22	ATRAP	49.0047°N, 16.4710°W	4848	05/27/2018
RRS James Cook	JC165	JC165#41	ATRAP	49.0047°N, 16.4710°W	4844	06/01/2018
RRS Discovery	DY103	DY103#10	ATRAP+	48.9434°N, 16.4847°W	4846	06/29/2019
RRS Discovery	DY103	DY103#24	ATRAP+	48.9439°N, 16.4850°W	4842	07/02/2019
RRS Discovery	DY130	DY130#29	ATRAP+	48.9931°N, 16.4372°W	4845	04/01/2021
RRS Discovery	DY130	DY130#54	ATRAP+	49.0212°N, 16.3974°W	4845	04/05/2021
RRS James Cook	JC247	JC247#12	ATRAP2+	49.0167°N, 16.3727°W	4845	05/09/2023
RRS James Cook	JC247	JC247#37	ATRAP2+	48.9812°N, 16.3672°W	4844	05/14/2023
RRS James Cook	JC263	JC263#47	ATRAP2+	49.0201°N, 16.3431°W	4846	06/01/2024

The holotype specimen was dissected and mounted onto permanent slides using polyvinyl-lactophenol stained with lignin pink. Illustrations were made using Olympus SZX10 and BX51 microscopes equipped with a camera lucida. Pencil drawings were scanned and inked digitally using Adobe Illustrator and a WACOM digitiser tablet (Coleman 2003, 2009). Some setae are omitted from the illustrations for clarity. Appendages of the right side are dissected and illustrated, unless otherwise stated.

In the descriptions and figures the following abbreviations were used: **A1, 2** = antenna 1, 2; **G1, 2** = gnathopod 1, 2; **H** = head; **LL** = lower lip; **Md** = mandible; **Mx1, 2** = maxilla 1, 2; **Mxp** = maxilliped; **P3–7** = pereopod 3–7; **U1–3** = uropod 1–3; **T** = telson; **l** = left; **r** = right; **m** = male; **f** = female.

Type material is deposited in the Natural History Museum, London (**NHMUK**), and additional material is held in the Discovery Collections, National Oceanography Centre, Southampton (**NOC**).

### DNA extraction, amplification, and sequencing

DNA was extracted from a pair of pleopods using a DNeasy Blood & Tissue Kit (QIAGEN) or QuickExtract (Lucigen) following the manufacturer’s instructions, with a final elution volume of 70 µl and 100 µl, respectively. Regions of 16S, cytochrome c oxidase subunit I (COI), 28S and Histone H3 (H3) were amplified with published primer sets (Cohen et al. 1998; Astrin and Stüben 2008; Corrigan et al. 2014; Lörz et al. 2018). The PCR mix for each reaction contained 10.5 μl of Red Taq DNA Polymerase 1.1× MasterMix (VWR), 0.5 μl of each primer (10 μM), and 1 μl of DNA template. Primers and PCR conditions are provided in Table 2.

**Table 2.** Primers and PCR conditions used to amplify target barcode genes in this study.

Gene	Primer	Direction	Sequence (5’–3’)	PCR program	Reference
**COI**	LCO1490-JJ	Forward	CHACWAAYCATAAAGATATYGG	1 × (2 min at 94 °C), 5 × (30 s at 94 °C, 90 s at 45 °C, 60 s at 72 °C), 35 × (30 s at 94 °C, 90 s at 51 °C, 60 s at 72 °C), 1 × (5 min at 74 °C)	Astrin and Stüben 2008
**COI**	HCO2198-JJ	Reverse	AWACTTCVGGRTGVCCAAARAATCA	1 × (2 min at 94 °C), 5 × (30 s at 94 °C, 90 s at 45 °C, 60 s at 72 °C), 35 × (30 s at 94 °C, 90 s at 51 °C, 60 s at 72 °C), 1 × (5 min at 74 °C)	Astrin and Stüben 2008
**16S**	16SFt_amp	Forward	GCRGTATIYTRACYGTGCTAAGG	1 × (2 min at 95 °C), 35 × (30 s at 95 °C, 30 s at 50 °C, 45 s at 72 °C), 1 × (5 min at 72 °C)	Lörz et al. 2018
**16S**	16SRt_amp	Reverse	CTGGCTTAAACCGRTYTGAACTC	1 × (2 min at 95 °C), 35 × (30 s at 95 °C, 30 s at 50 °C, 45 s at 72 °C), 1 × (5 min at 72 °C)	Lörz et al. 2018
**28S**	28Sftw	Forward	AGAAACTAACMAGGATTCCYYTAGTA	1 × (2 min at 95 °C), 35 × (40 s at 94 °C, 40 s at 50 °C, 40 s at 72 °C), 1 × (10 min at 72 °C)	Corrigan et al. 2014
**28S**	28Srtw	Reverse	ACTTTCCCTCAYGGTACTTGT	1 × (2 min at 95 °C), 35 × (40 s at 94 °C, 40 s at 50 °C, 40 s at 72 °C), 1 × (10 min at 72 °C)	Corrigan et al. 2014
**H3**	HisH3f	Forward	AAATAGCYCGTACYAAGCAGAC	1 × (2 min at 95 °C), 35 × (40 s at 94 °C, 40 s at 45 °C, 40 s at 72 °C), 1 × (10 min at 72 °C)	Corrigan et al. 2014
**H3**	HisH3r	Reverse	ATTGAATRTCYTTGGGCATGAT	1 × (2 min at 95 °C), 35 × (40 s at 94 °C, 40 s at 45 °C, 40 s at 72 °C), 1 × (10 min at 72 °C)	Corrigan et al. 2014

PCR products were purified using 4 μl of VMR ExoCleanUp FAST (VWR) per 10 μl PCR product before sequencing. The cleaning program was set to 37 °C × 5 min/ 80 °C × 10 min. Bidirectional Sanger sequencing was performed by Eurofins Genomics using the same primers as the PCR. For each gene fragment contigs were *de novo* assembled by aligning both forward and reverse sequences with default settings and trimming the primer sequences using Geneious v. 7.0.6 (Kearse et al. 2012). Chromatograms were visually inspected, with ambiguous base calls corrected manually. Chromatograms were then translated to amino acids (COI and H3 only) to look for stop codons and then compared against public databases to remove unwanted sequences. It is of note that we also attempted to sequence PCR products from 18S rDNA fragments using 18SA (Medlin et al. 1988) and 18SB (Nygren and Sundberg 2003) primer pairs. However, after sequencing, gene fragment contigs could not be assembled from forward and reverse sequences due to poor read quality.

### Phylogenetic inference and genetic divergence

Phylogenetic inference was performed to investigate the phylogenetic placement of *Caeconyx papso* sp. nov. with other members of the family Uristidae and morphologically allied members of the family Tryphosidae. Sequences for 16S, COI, H3, and 28S genes from members of the family Uristidae and Tryphosidae were downloaded from NCBI and BOLD systems and used for phylogenetic inference. Information regarding the source of each sequence is given in Suppl. material 1. Sequence alignments for each marker were conducted using MAFFT v. 7, employing the FFT-NS-I strategy (Katoh et al. 2019).

Phylogenetic inference was conducted separately for both a COI sequence dataset and for a concatenated multilocus dataset (COI, H3, 16S, 28S) using a maximum-likelihood and Bayesian inference approach. Maximum-likelihood analyses were conducted in IQ-TREE (Wong et al. 2025) using the best substitution models and partitioning scheme selected by ModelFinder (Kalyaanamoorthy et al. 2017) under the MFP+MERGE option, with rapid Bootstrap, and node support estimated from 1000 bootstrap replicates. Bayesian inference analyses were conducted in MrBayes (Huelsenbeck and Ronquist 2001) under partitioned models using the best substitution models selected above.

Corrected intraspecific and interspecific genetic divergence (K2P; Kimura 1980) were estimated for COI in MEGA X (Kumar et al. 2018). Variance was estimated through 500 bootstraps.

## Results

### Systematics

**Order Amphipoda Latreille, 1816**

#### Suborder Amphilochidea Boeck, 1871

**Superfamily Lysianassoidea Dana, 1849**

**Family Uristidae Hurley, 1963**

##### Genus Caeconyx

Taxon classificationAnimaliaAmphipodaUristidae

Barnard & Karaman, 1991

61D49A0F-752B-52D6-9D37-46F6C7CC38F5

Caeconyx Barnard & Karaman, 1991: 473—Lowry and Kilgallen 2014: 12 (fig. 3).

###### Type species.

*Hoplonyx caeculus* G.O. Sars, 1891, original designation.

###### Included species.

*Caeconyx* contains two species: *Caeconyx caeculus* G.O. Sars, 1891; *C. papso* sp. nov.

###### Diagnosis.

(after Barnard and Karaman 1991 and Lowry and Kilgallen 2014) Antenna 1 peduncle article 1 without anterodistal lobe; accessory flagellum forming cap covering callynophore. Antenna 2 without brush setae. Mandible molar setose with vestigial triturating surface (*C. caeculus*) or an asymmetrically reduced column, with a distally triturative surface (*C. papso* sp. nov.). Maxilla 1 outer plate with a well-developed 7/4 crown. Maxilla 2 inner plate slightly shorter than outer plate. Gnathopod 1 subchelate; coxa 1 large, about as long as coxa 2, subrectangular with straight anterior margin; ischium short (length less than 2× breadth); carpus long (length 2–4× breadth); propodus margins subparallel. Uropod 2 inner ramus not constricted. Telson deeply cleft.

###### Remarks.

According to Lowry and Kilgallen (2014), *Caeconyx* is most similar to the uristid genera *Anonyx* and *Tmetonyx*. *Caeconyx* differs from *Anonyx* in the mandibular palp attachment (level with molar in *Anonyx*, distal to molar in *Caeconyx*), and the margins of the gnathopod 1 propodus which are tapering in *Anonyx* (subparallel in *Caeconyx*). *Caeconyx* differs from *Tmetonyx* in having an accessory flagellum forming a cap (lacking in *Tmetonyx*), acute lateral cephalic lobes (rounded in *Tmetonyx*), and a short gnathopod 1 ischium (long in *Tmetonyx*). The mandibular molar is setose with a vestigial triturating surface in both *Anonyx* and *Caeconyx*, (reduced column with a moderate triturative surface in *Tmetonyx*).

###### Distribution.

Northeast Atlantic Ocean and Mediterranean Sea. Iceland to Trondheimsfjord and the Mediterranean Sea, 150–1095 m (*C. caeculus*); Porcupine Abyssal Plain, 4850 m (*C. papso* sp. nov.).

##### Caeconyx papso sp. nov.

Taxon classificationAnimaliaAmphipodaUristidae

EB1AAF64-AFD2-527A-9A3B-FFC275AF2A24

https://zoobank.org/BAABFD6E-0D34-4178-999D-2C92F80732EF

Figs 1, 2, 3, 4, 5

###### Type material.

***Holotype***: Atlantic Ocean • mature female (setose oostegites apparent), 8.93 mm; carcass and nine slides; Porcupine Abyssal Plain; 48.9683°N, 16.4155°W; depth 4845 m; 19/3/1997; BENGAL, RRS Discovery, Cruise D226, Station 13077#35, Ptraps (NIOZ trap); NHM UK 2026.446 (specimen 13077#35_Trap1_3).

***Paratypes***: Atlantic Ocean • mature male (penes apparent), 8.32 mm; carcass; Porcupine Abyssal Plain; 48.9683°N, 16.4155°W; depth 4845 m; 19/3/1997; BENGAL, RRS Discovery, Cruise D226, Station 13077#35, Ptraps (NIOZ trap); NHM UK 2026:447 (specimen 13077#35_Trap1_1) • mature female (setose oostegites apparent), 9.07 mm; carcass; Porcupine Abyssal Plain; 48.9683°N, 16.4155°W; depth 4845 m; 3/19/1997; BENGAL, RRS Discovery, Cruise D226, Station 13077#35, Ptraps (NIOZ trap); NHM UK 2026:448 (specimen 13077#35_Trap1_2) • mature male (penes apparent), 9.29 mm; carcass; Porcupine Abyssal Plain; 48.9683°N, 16.4155°W; depth 4845 m; 19/3/1997; BENGAL, RRS Discovery, Cruise D226, Station 13077#35, Ptraps (NIOZ trap); NHM UK 2026:449 (specimen 13077#35_Trap2_2) • mature female (setose oostegites apparent), 8.47 mm; carcass; Porcupine Abyssal Plain; 49.0047°N, 16.4710°W; depth 4844 m; 1/6/2018; BENGAL, RRS James Cook, Cruise JC165, Station JC165#41, Atrap; DISCOLL-JC165-41-T1-1 (specimen JC165#41_T1_1), COI (PZ145156), H3 (PZ148823), 16S (PZ147694), 28S (PZ147690).

###### Other material.

Atlantic Ocean • Porcupine Abyssal Plain, several stations (Table 3).

**Table 3.** Material examined and genetic sequence information. n.d. = not determined either owing to damaged specimen or too small to determine.

Specimen number	Type status	Sex	Size (mm)	Catalogue number	Sample /station number	COI	16S	28S	H3
***Caeconyx papso* sp. nov**.									
52216#5_Trap2_1	–	Female	6.52	–	52216#5	–	–	–	–
53201#25_1	–	n.d.	2.66	–	53201#25	–	–	–	–
53201#25_2	–	n.d.	2.88	–	53201#25	–	–	–	–
13077#04_Trap1_1	–	Female	7.69	–	13077#04	–	–	–	–
13077#04_Trap1_2	–	Female	7.16	–	13077#04	–	–	–	–
13077#04_Trap1_3	–	Male	9.22	–	13077#04	–	–	–	–
13077#04_Trap1_4	–	n.d.	4.51	–	13077#04	–	–	–	–
13077#35_Trap1_1	Paratype	Male	8.32	NHM UK 2026:447	13077#35	–	–	–	–
13077#35_Trap1_2	Paratype	Female	9.07	NHM UK 2026:448	13077#35	–	–	–	–
13077#35_Trap1_3	Holotype	Female	8.93	NHM UK 2026:446	13077#35	–	–	–	–
13077#35_Trap1_4	–	n.d.	9.69	–	13077#35	–	–	–	–
13077#35_Trap1_5	–	n.d.	n.d.	–	13077#35	–	–	–	–
13077#35_Trap2_1	–	Male	7.37	–	13077#35	–	–	–	–
13077#35_Trap2_2	Paratype	Male	9.28	NHM UK 2026:449	13077#35	–	–	–	–
13077#35_Trap2_3	–	n.d.	9.40	–	13077#35	–	–	–	–
13077#35_Trap3_1	–	Female	8.22	–	13077#35	–	–	–	–
13077#35_Trap3_2	–	Female	8.35	–	13077#35	–	–	–	–
13077#35_Trap3_3	–	Male	9.07	–	13077#35	–	–	–	–
13077#92_1	–	Male	7.79	–	13077#92	–	–	–	–
13078#3_1	–	n.d.	n.d.	–	13078#3	–	–	–	–
JC062#103_T1_1	–	Male	7.90	–	JC062#103	–	–	–	–
JC062#103_T1_2	–	Male	8.05	–	JC062#103	–	–	–	–
JC062#103_T1_3	–	Male	6.48	–	JC062#103	–	–	–	–
JC062#103_T1_4	–	Male	6.96	–	JC062#103	–	–	–	–
JC062#103_T1_5	–	Female	6.73	–	JC062#103	–	–	–	–
JC062#103_T1_6	–	n.d.	4.45	–	JC062#103	–	–	–	–
JC062#103_B1_1	–	Male	7.46	–	JC062#103	–	–	–	–
JC062#103_B2_1	–	Female	5.79	–	JC062#103	–	–	–	–
JC062#103_B2_2	–	Male	8.24	–	JC062#103	–	–	–	–
JC071#34_3’_1	–	Male	8.65	–	JC071#34	–	–	–	–
DY077#61_B1_1	–	Male	6.48	–	DY077#61	–	–	–	–
DY077#83_T2_1	–	Female	7.11	DISCOLL-DY077-83-T2-1	DY077#83	PZ145162			–
DY077#83_B2_1	–	Male	6.87	–	DY077#83	–	–	–	–
JC165#22_T2_1	–	Female	8.16	–	JC165#22	–	–	–	–
JC165#22_B2_1	–	Male	7.93	DISCOLL-JC165-22-B2-1	JC165#22	PZ145158	–	–	–
JC165#41_T1_1	Paratype	Female	8.47	DISCOLL-JC165-41-T1-1	JC165#41	PZ145156	PZ147694	PZ147690	PZ148823
JC165#41_T1_2	–	Male	7.75	–	JC165#41	–	–	–	–
JC165#41_T1_3	–	Male	7.49	–	JC165#41	–	–	–	–
JC165#41_T1_4	–	Female	8.08	–	JC165#41	–	–	–	–
JC165#41_T1_5	–	Male	9.02	DISCOLL-JC165-41-T1-5	JC165#41	PZ145157	PZ147695	PZ147691	PZ148824
JC165#41_T1_6	–	Male	7.27	–	JC165#41	–	–	–	–
JC165#41_T1_7	–	Male	5.71	DISCOLL-JC165-41-T1-7	JC165#41	PZ145159	–	–	–
JC165#41_T2_1	–	Male	5.38	–	JC165#41	–	–	–	–
JC165#41_B2_1	–	Male	7.88	–	JC165#41	–	–	–	–
JC165#41_B2_2	–	n.d.	n.d.	–	JC165#41	–	–	–	–
DY103#10_B1_1	–	n.d.	4.42	DISCOLL-DY103-10-B1-1	DY103#10	PZ145163	–	–	–
DY103#10_B2_1	–	Female	7.52	–	DY103#10	–	–	–	–
DY103#10_B2_2	–	Male	8.98	–	DY103#10	–	–	–	–
DY103#10_B2_3	–	n.d.	5.87	–	DY103#10	–	–	–	–
DY103#24_B2_1	–	n.d.	5.88	–	DY103#24	–	–	–	–
DY130#29_T2_1	–	Male	7.76	–	DY130#29	–	–	–	–
DY130#54_B1_1	–	Female	n.d.	–	DY130#54	–	–	–	–
JC247#37_B1_1	–	Female	9.09	DISCOLL-JC247-37-B1-1	JC247#37	PZ145160	–	–	–
JC263#47_B1_1	–	Male	8.20	DISCOLL-JC263-47-B1-1	JC263#47	PZ145161	–	–	–
JC263#47_B1_2	–	Male	4.90	–	JC263#47	–	–	–	–
***Centromedon* sp. DISCOLL_PAP_JC247#12**									
JC247#12_B1_1	–	n.d.	5.02	DISCOLL-JC247-12-B1-1	JC247#12	PZ145164	PZ147693	PZ147692	PZ148825

###### Type locality.

Abyssal Atlantic Ocean, Porcupine Abyssal Plain, 48.9683°N, 16.4155°W; depth 4845 m.

###### Diagnosis.

Head with broadly triangular sub-acute eye lobe. Epimeron 1 posterodistal margin convex and broadly rounded. P3 and P4 propodus not slender or elongated (length ~1.1× carpus for P3, length ~1.3× carpus for P4).

###### Description.

Based on holotype mature female (setose oostegites), 8.93 mm, NHM UK 2026.446.

**Figure 1.**
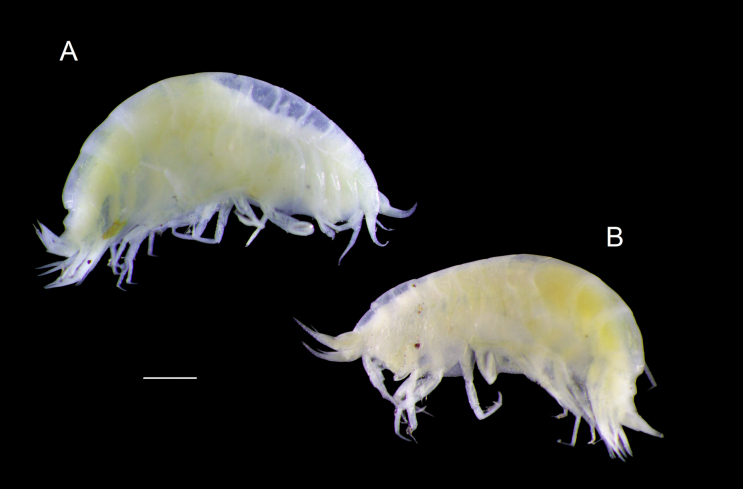
*Caeconyx papso* sp. nov. **A**. Mature female, 8.93 mm, holotype: NHM UK 2026.446; **B**. Mature male, 8.32 mm; paratype: NHM UK 2026:447. Scale bar: 1 mm.

**Figure 2.**
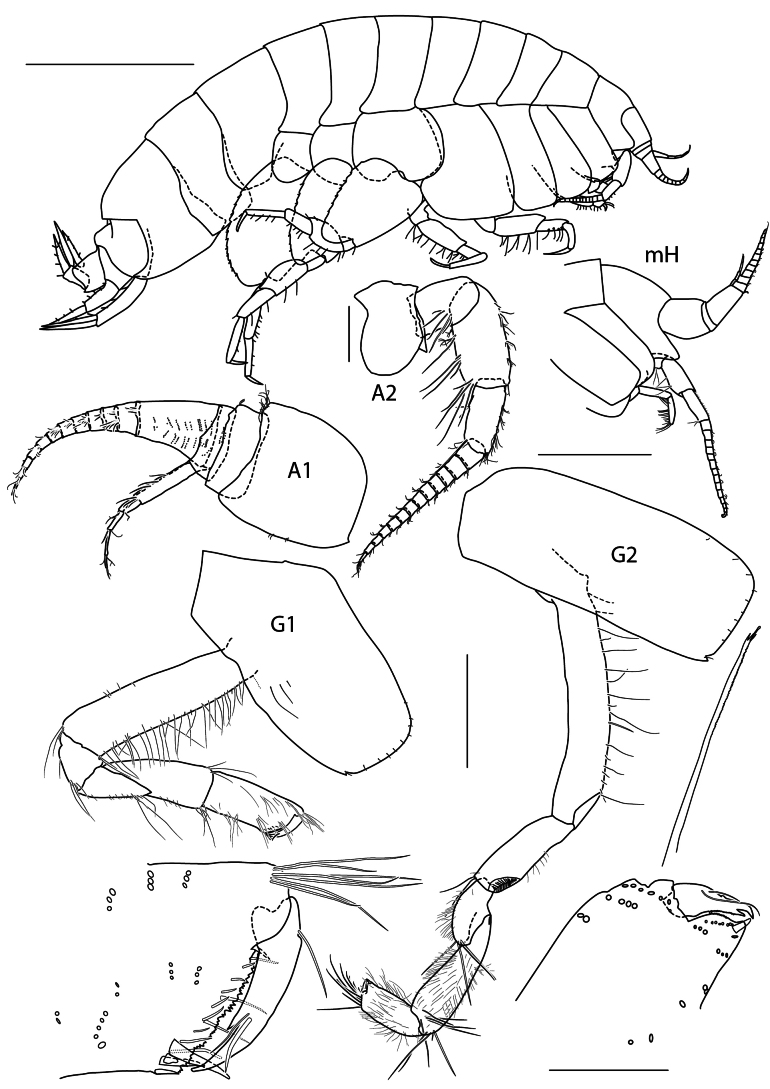
*Caeconyx papso* sp. nov., mature female, 8.93 mm, NHM UK 2026.446; mH = head of Paratype mature male, 8.32 mm, NHM UK 2026:447. Scale bars: 2 mm (habitus); 0.2 mm (A1, A2); 0.5 mm (G1, G2); 1 mm (mH).

***Body*** (Figs 1, 2): ***Pereonites 1–7*** deeper than broad. ***Pleonite 3*** with a rounded, posterodorsal elevation overhanging urosomite 1. ***Urosomite 1*** with a dorsal concavity in front of prominent rounded boss. ***Urosomite 2*** very short, telescoped under urosomite 1. ***Epimeron 1*** subquadrate, posterodistal margin convex, broadly rounded. ***Epimeron 2*** subquadrate, anterodistal corner rounded, distal margin slightly convex, posterodistal corner not produced, posterior margin straight. ***Epimeron 3*** ventral margin straight, posterodistal corner produced into a small tooth. ***Coxae 1–4*** longer than corresponding pereonites, coxa 1 not shortened.

***Head*** (Figs 1, 2): slightly shorter than pereonite 1, length equal to pereonite 2; rostrum short, reaching halfway along lateral cephalic lobe. ***Lateral cephalic lobe*** broadly triangular, subacute. ***Eye***: not present. ***Antenna 1*** short, length 0.14× body; peduncular article 1 dilated, length 1.13× width; peduncular articles 2 and 3 very short; flagellum 11-articulate, first article of flagellum with two-field callynophore; accessory flagellum five-articulate, first article forming cap, calceoli absent. ***Antenna 2*** slightly longer than antenna 1, geniculate between articles 3 and 4, gland cone present; peduncular article 4 longer and broader than 5, with three groups of long posteromedial setae; flagellum 14-articulate, calceoli absent.

**Figure 3.**
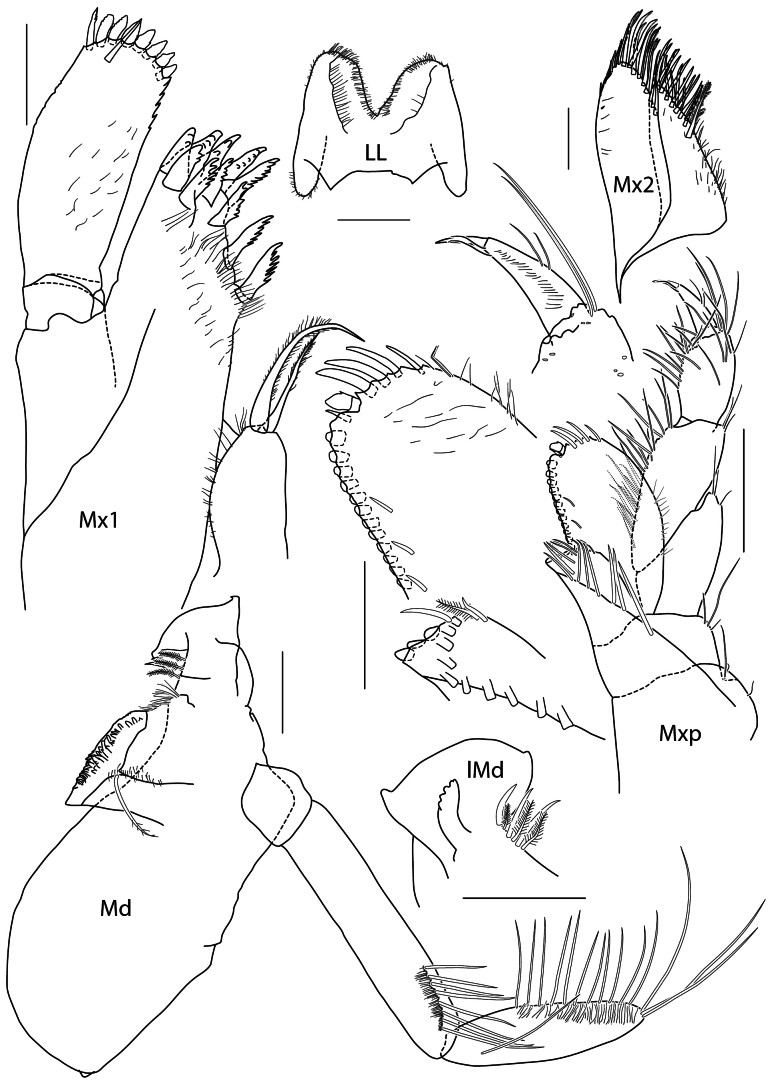
*Caeconyx papso* sp. nov., mature female, 8.93 mm, NHM UK 2026.446. Scale bars: 0.1 mm (Mx1, 2, Md, lMd); 0.2 mm (LL, Mxp).

***Mouthparts*** (Figs 1, 2, 3): ***epistome*** straight, smooth, slightly dominant. ***Upper lip*** (Fig. 2): broadly rounded. ***Mandible*** (Fig. 3): incisor convex and widened, with tooth at anterodistal and posterodistal corners; left lacinia mobilis five-dentate, right lacking; accessory spine row with three robust setae interspersed with three plumose setae; molar an asymmetrically reduced column, distally triturative with long plumose seta; palp level with molar, article 2 1.34× length of article 3, with eight A2-setae, article 3 ovate, with 12 D3-pectinate setae and three E3-setae. ***Lower lip*** (Fig. 3): outer plates separated, symmetrical, setose marginally and apically, inner plates absent. ***Maxilla 1*** (Fig. 3): inner plate with two plumose setae apically; outer plate broad, with 11 spine-teeth in 7/4 crown arrangement, ST1–2 two-cuspidate, ST3 three-cuspidate, ST4 two-cuspidate, ST5 five-cuspidate, ST6 seven-cuspidate, ST7 six-cuspidate, ST7 displaced from ST6, STA–B four-cuspidate, STC-D five-cuspidate; palp long, slender, two-articulate, article 1 short, article 2 with seven short conical spines and one flag seta on apical margin, and one sub-apical robust seta. ***Maxilla 2*** (Fig. 3): inner plate slightly shorter and narrower than outer, tapering distally; inner plate with two rows of pectinate setae on medial margin and one discontiguous plumose seta; outer plate with a row of pectinate setae on the medial and apical margins. ***Maxilliped*** (Fig. 3): inner plate subrectangular, reaching halfway up the outer plate, distal margin with three nodular spines in a row and three setae, 11 long plumose setae in a curved row from apical margin to medial edge; outer plate subovate, reaching the distal end of palp article 2, with four strong apical robust setae and one large apical nodular spine with a cusp, 12 medial nodular spines; palp ordinary, setose medially.

**Figure 4.**
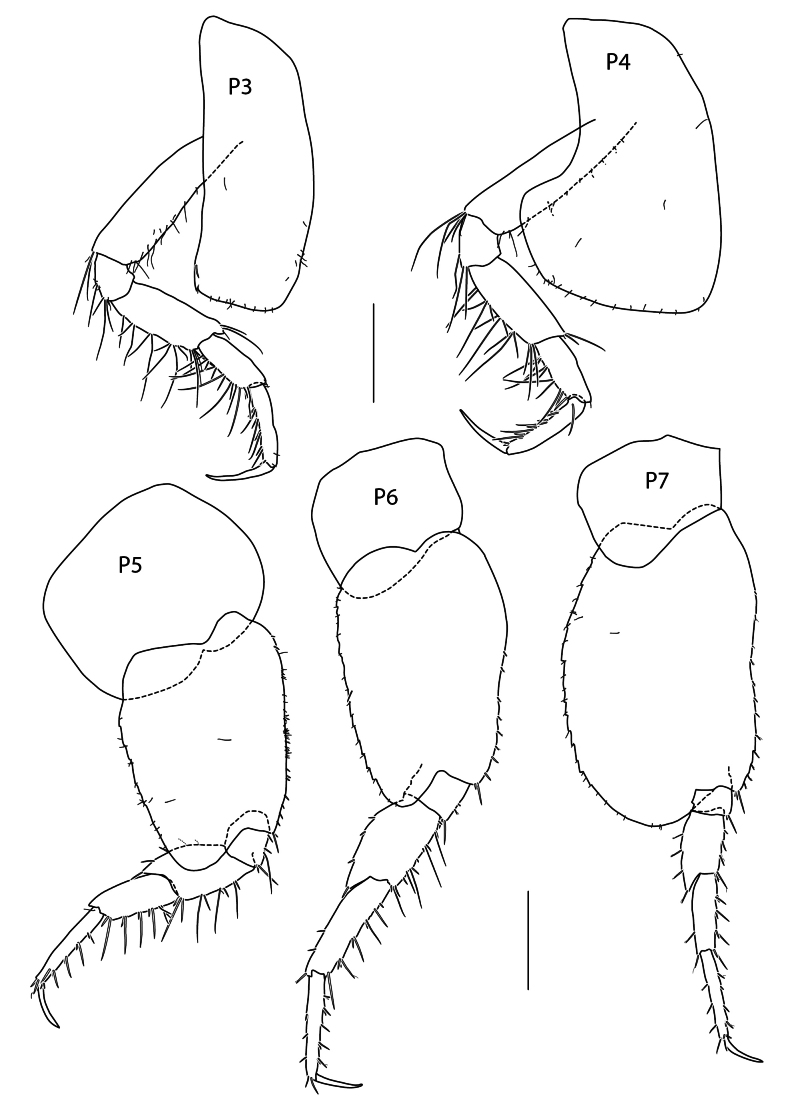
*Caeconyx papso* sp. nov., mature female, 8.93 mm, NHM UK 2026.446. Scale bars: 0.5 mm (P3–P7).

***Pereon*: *gnathopod 1*** (Fig. 2): coxa subrectangular, length 2× width, anterior margin straight, ventral margin with small notch and seta; basis, anterior margin with a row of numerous long setae proximally and long setae distally; ischium shorter than merus; carpus length 1.17× propodus; propodus subchelate, subrectangular, margins subparallel, palm acute, palmar corner defined by one large robust seta and two smaller robust setae; dactylus almost reaching palmar corner, with subterminal tooth and two setae ventrally and a single seta dorsally. ***Gnathopod 2*** (Fig. 2): coxa subrectangular, length 2.47× width, ventral margin with small notch and seta; basis narrow, length 6.25× width, anterior margin setose; ischium longer than merus; carpus length 1.8× propodus; propodus minutely chelate, subrectangular, covered by fine setae, with anterodistal groups of long tridentate setae (Fig. 2), palm short, obtuse, palmar corner defined by a robust seta; dactylus meeting palmar corner, with a subterminal tooth and two fine setae dorsally. ***Pereopod 3*** (Fig. 4): coxa subrectangular, with anterior margin slightly convex, ventral margin with small notch and seta, length 2.5× width; merus longer and broader than carpus, posterior margins setose; propodus longer and narrower than carpus, posterior margin setose; dactylus slightly curved, shorter than propodus. ***Pereopod 4*** (Fig. 4): coxa length 1.45× width, anterior margin convex, posterior margin deeply excavate proximally, with a broadly rounded posterodistal lobe, ventral margin broadly rounded; remaining pereopod articles as in pereopod 3. ***Pereopod 5*** (Fig. 4): coxa aequilobate; basis length 1.37× width, anterior and posterior margins spinose, posterior margin convex, with broadly rounded posterodistal lobe; merus expanded, subequal to carpus, anterior margin spinose; carpus broad, anterior margin spinose; propodus narrow, longer than carpus, anterior margin spinose; dactylus long, curved, shorter than propodus. ***Pereopod 6*** (Fig. 4): coxa slightly posterolobate, subquadrate; basis, length 1.5× width, anterior and posterior margins spinose, anterior margin slightly convex with small robust setae distally, posterior margin convex, with broadly rounded posterodistal lobe reaching merus; carpus longer than merus and propodus longer than carpus; dactylus long, curved, shorter than propodus. ***Pereopod 7*** (Fig. 4): coxa subrectangular, posterolobate; basis broadly expanded, length 1.4× width, anterior and posterior margins spinose, posterior margin convex, with broadly rounded posterodistal lobe reaching merus; distal articles as in pereopod 6.

**Figure 5.**
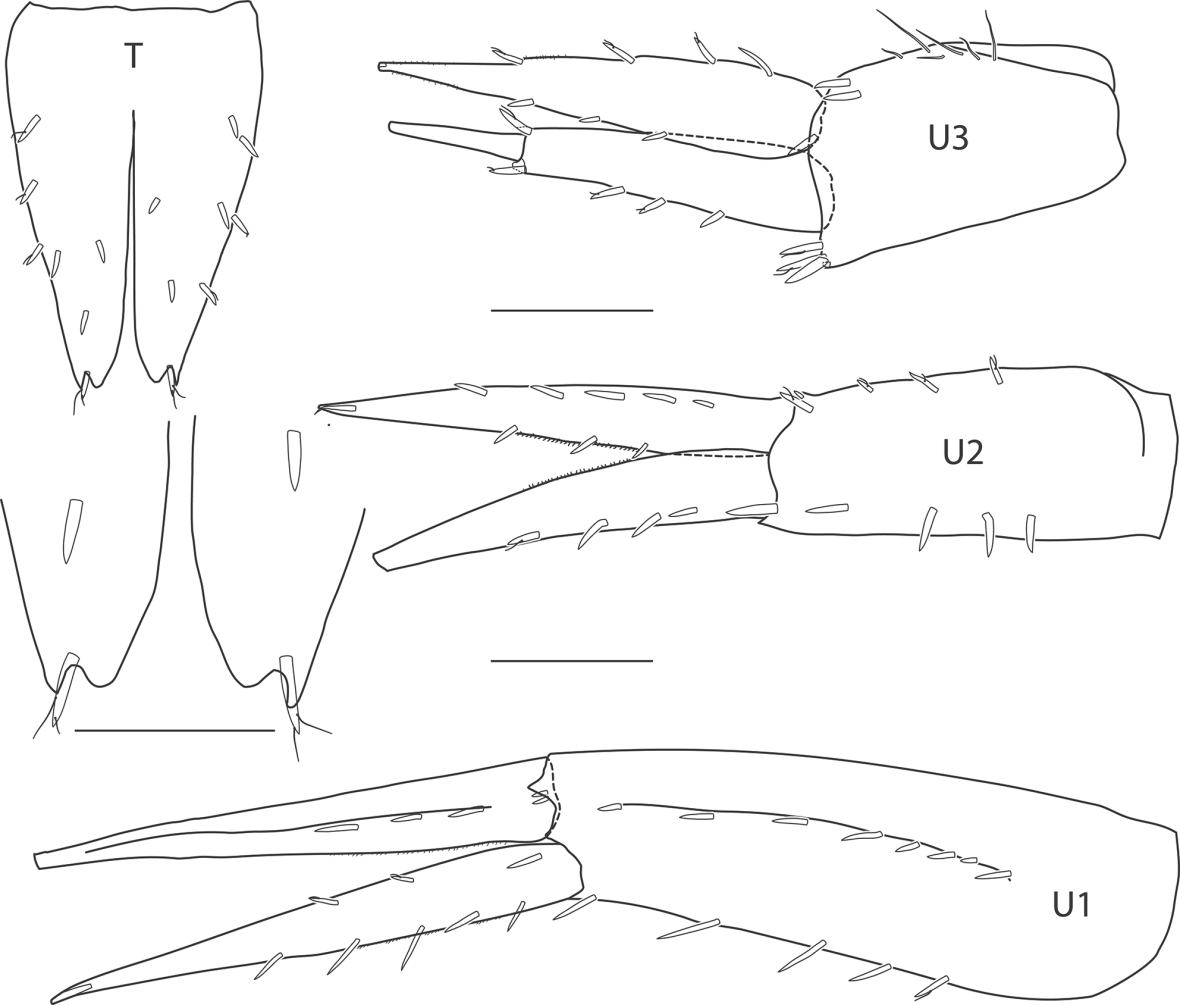
*Caeconyx papso* sp. nov., mature female, 8.93 mm. NHM UK 2026.446. Scale bars: 0.2 mm (U1–U3, T).

***Urosome*** (Figs 1, 5): ***uropod 1*** peduncle slightly longer than rami, dorsolateral and dorsomedial margins spinose, with eight and five spines, respectively; rami lanceolate, apically with a single inset spine, inner ramus slightly shorter than outer, dorsolateral and dorsomedial margins with three and five spines, respectively; outer ramus, dorsolateral margin with three spines, dorsomedial margin lacking spines. ***Uropod 2*** peduncle shorter than outer ramus, dorsolateral margin with four robust setae and one apical robust seta, dorsomedial margin with three robust seta and two apical robust setae; rami lanceolate with apical inset spine, inner ramus dorsolateral and dorsomedial margins with three and five robust setae, respectively; outer ramus, dorsolateral margin with four robust setae. ***Uropod 3***, peduncle 0.66× length of biarticulate outer ramus, with three distoventral robust setae and two robust setae dorsodistally; second article of outer ramus damaged, 0.4× length of article 1, article 1 with three dorsolateral robust setae, one dorsolateral robust seta and small tooth apically, and one dorsomedial seta and small tooth apically; inner ramus subequal to outer ramus, with three dorsolateral and four dorsomedial robust setae. ***Telson*** ~ 1.5× longer than wide, deeply cleft 73%, lobes tapering distally with 6–7 robust setae per lobe. Apices notched inset with a single robust seta with two setules.

***Oostegites*** present on gnathopod 2 and pereopods 3–5, setose, slender.

***Gills*** present on gnathopod 2 and pereopods 3–7.

###### Sexual dimorphism.

(Fig. 1) Males present calceoli on antenna 1 flagellum articles 8–11 and on all flagellum articles on antenna 2. The number of articles on the antenna 1 primary and accessory flagellum, as well as the antenna 2 flagellum are identical to that of the female. The head lobe appears broadly triangular and subacute on all examined male and female specimens of *C. papso* sp. nov. This is in contrast to the shape of the head lobe in *C. caeculus*, which may be sexually dimorphic. The figures of G.O. Sars (1891) of a 5 mm female show a strong narrowly triangular, acute head lobe, whereas the 3.7 mm male illustrated by Lowry and Kilgallen (2014) possesses a less narrowly triangular, more subacute head lobe.

###### Etymology.

The species is named in honour of the Porcupine Abyssal Plain Sustained Observatory (PAP-SO), recognising the four decades of multidisciplinary scientific research conducted at the site. *Caeconyx papso* sp. nov. was first identified as a potential new species (provisionally assigned to the genus *Paracentromedon*) by one of the authors (MHT) in 1985 (cruise CH6A/85), marking the beginning of the PAP-SO scavenging amphipod time series. Since then, 54 individuals of *Caeconyx papso* sp. nov. have been identified over the 40 years of scavenging amphipod trap work carried out at PAP-SO, with the most recent specimen identified in 2024. The species epithet is used as a noun in apposition.

###### Remarks.

The new species is very similar to its congener *C. caeculus* and can be distinguished from that species by the characters indicated in the diagnosis. The two species can be differentiated from each other by the eye lobe, which is broadly triangular and sub-acute in both males and females of *C. papso* sp. nov., differing from the much more narrowly triangular and acute eye lobe seen in females of *C. caeculus*; the posterodistal margin of epimeron 1, which is convex and broadly rounded in *C. papso* sp. nov. but straight in *C. caeculus*; and the form of the propodi of pereopods 3 and 4 which are not slender or elongate in *C. papso* sp. nov. (length 1.13× carpus for P3; 1.29× carpus for P4), and clearly differ from the elongate, slender propodi of P3 and P4 in *C. caeculus* (length 1.47× carpus for P3; 2.0× carpus for P4). Additional characters that also separate the two species, include the accessory flagellum (5-articulate in *C. papso* sp. nov. versus 4-articulate in *C. caeculus*); the antenna 2 flagellum (14-articulate in *C. papso* sp. nov. versus 8-articulate in *C. caeculus*); the palmar angle of gnathopod 1 which is less acute in *C. papso* sp. nov. than in *C. caeculus*; and the coxa of pereopod 4 which differs in shape between the two species, with a straighter distal margin in *C. caeculus* compared to the broadly rounded distal corner in *C. papso* sp. nov.

###### Distribution.

Abyssal Atlantic Ocean, Porcupine Abyssal Plain, 4677–4848 m.

###### Molecular data.

Sequences of one of the paratypes and additional individuals of *Caeconyx papso* sp. nov. are deposited in GenBank with the following accession numbers, COI: PZ145156–PZ145164; 28S: PZ147690–PZ147692; 16S: PZ147693–PZ147695; H3: PZ148823–PZ148825.

###### Phylogenetic inference.

**Figure 6.**
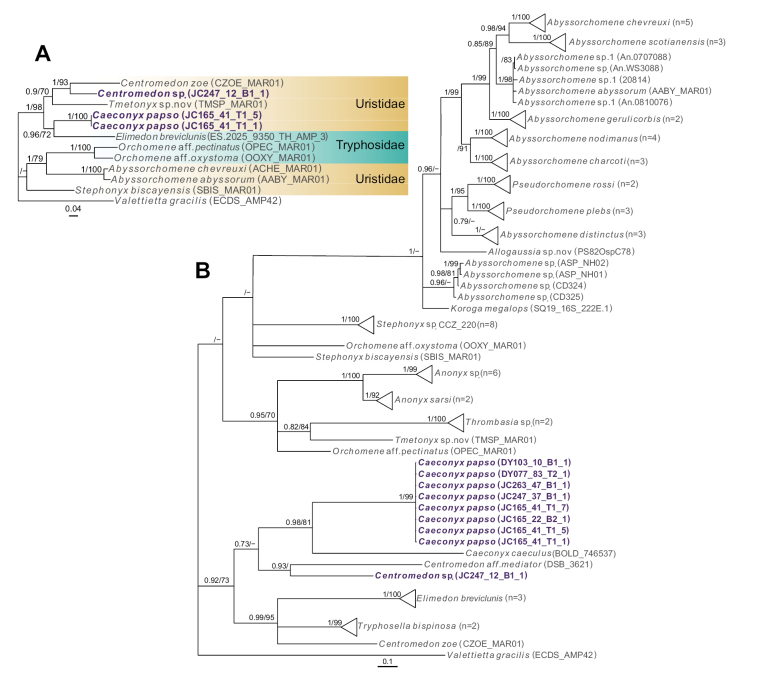
Phylogenetic reconstructions of *Caeconyx papso* sp. nov. in relation to other members of the Uristidae, and morphologically allied taxa within the Tryphosidae, based on a concatenated dataset of COI, H3, 28S, and 16S genetic markers (**A**) and COI only (**B**). Median consensus trees (Bayesian inference, BI) showing posterior probability (PP) and bootstrap support values (BS) from BI and maximum-likelihood analyses, respectively, at each node. Only values higher or equal to 0.7 or 70 for PP or BS are indicated.

Two phylogenetic reconstructions of comparative taxa are presented from morphologically allied taxa with target sequences available in GenBank: one based on a concatenated dataset of the COI, H3, 28S, and 16S genetic markers (Fig. 6a), and the other based solely on COI (Fig. 6b).

Phylogenetic trees estimated from both maximum likelihood (ML) and Bayesian inference (BI) for both COI and concatenated datasets recovered all specimens of *Caeconyx papso* sp. nov. in a well-supported monophyletic clade. Intraspecific genetic divergence for COI was only 0.03% for *C. papso* sp. nov. (Table 4). Sequences of *C. papso* sp. nov. and a sequence of its congener *C. caeculus* were recovered in a (well-supported for BI) monophyletic clade in both phylogenies (posterior probability (PP) = 0.98, bootstrap support (BS) = 81). Interspecific genetic divergence using COI between *C. papso* sp. nov. and *C. caeculus* was 22%. Both ML and BI phylogenetic analyses suggested genetic affinities of *C. papso* sp. nov. with the tryphosid genera *Elimedon* J.L. Barnard, 1962 (*Elimedon breviclunis* Horton et al., 2026a) and *Tryphosella* (*Tryphosella bispinosa* Schellenberg, 1931), as well as the uristid genera *Tmetonyx*Stebbing 1906 (*Tmetonyx* sp. nov. TMSP_MAR01) and *Centromedon* G.O. Sars, 1891 (*Centromedon zoe* Horton & Thurston, 2011) and *Centromedon* sp. DISCOLL_PAP_JC247#12).

**Table 4.** Corrected (K2P) genetic distances (%) for *Caeconyx papso* sp. nov. and phylogenetically related species. Intraspecific genetic distances: n.d. = not determined due to only a single sequence being available.

	*Tmetonyx* sp. nov.	*Tryphosella bispinosa*	*Centromedon zoe*	*Elimedon breviclunis*	*Caeconyx papso* sp. nov.	*Centromedon* sp.	*Centromedon aff. mediator*	*Caeconyx caeculus*
***Tmetonyx* sp. nov**.	n.d.	–	–	–	–	–	–	–
***Tryphosella bispinosa***	24.13	0.176	–	–	–	–	–	–
***Centromedon zoe***	26.45	19.78	n.d.	–	–	–	–	–
***Elimedon breviclunis***	28.77	21.63	23.55	–	–	–	–	–
***Caeconyx papso* sp. nov**.	26.86	24.22	23.7	22.2	0.033	–	–	–
***Centromedon* sp. DISCOLL_PAP_JC247#12**	26.84	22.79	28.14	24.16	20.41	n.d.	–	–
***Centromedon aff. mediator***	25.11	24.56	24.68	25.57	21.65	20.31	n.d.	–
***Caeconyx caeculus***	29.94	24.47	31.64	27.19	22.18	21.32	24.78	n.d.

In accordance with previous phylogenetic analyses (Corrigan et al. 2014; Stewart et al. 2024), phylogenetic inference based on the concatenated dataset demonstrated the paraphyly of the families Tryphosidae and Uristidae (Fig. 6a). However, it is recognised that the number of taxa used in this analysis is low compared with the known diversity in these two lysianassidiran families (there are 27 genera and 190 species in the Uristidae, and 43 genera and 389 species in the Tryphosidae; Horton et al. 2023, 2026b). The confidence in such analyses will only be improved only by the inclusion of a greater number of comparative taxa and more genetic markers.

During the examination of *Caeconyx papso* sp. nov. specimens, a specimen previously identified as *Paracentromedon* sp. DISCOLL_PAP_52216 was re-identified as *Centromedon* sp. according to the short distal articles of pereopod 7. Bayesian inference phylogeny based on the concatenated dataset supported the assignment of this specimen to the genus *Centromedon*, forming a well-supported clade with *Centromedon zoe* (Fig. 6a). This specimen has therefore been given the temporary name *Centromedon* sp. DISCOLL_PAP_JC247#12, awaiting further analysis of additional specimens.

### Key to the species of *Caeconyx*

**Table:** 

1	Head with narrowly triangular, acute eye lobe, epimeron 1 posterodistal margin straight, P3 and P4 propodus long and slender (length ~1.5× carpus for P3, length 2× carpus for P4)	***C. caeculus***
–	Head with broadly triangular sub-acute eye lobe, epimeron 1 posterodistal margin convex and broadly rounded, P3 and P4 propodus not slender or elongated (length ~1.1× carpus for P3, length ~1.3× carpus for P4)	***C. papso* sp. nov**.

## Supplementary Material

XML Treatment for Caeconyx

XML Treatment for Caeconyx papso

## Additional information

### Artificial Intelligence (AI) use

The authors accept full responsibility for the content of the manuscript, including the disclosure of any use of AI.

No AI tools were used in the preparation of this manuscript.

### Author ORCIDs

B. Walker https://orcid.org/0009-0008-9713-5652

M.H. Thurston https://orcid.org/0000-0001-5627-616X

G. Bribiesca-Contreras https://orcid.org/0000-0001-8163-8724

T. Horton https://orcid.org/0000-0003-4250-1068

### Data availability

All of the data that support the findings of this study are available in the main text or Supplementary Information.

## Supplementary materials

Supplementary material 1 Phylogenetic data Data type xlsx Explanation note Accession numbers and references for sequences used in phylogenetic analyses. https://binary.pensoft.net/file/1682264This dataset is made available under the Open Database License (http://opendatacommons.org/licenses/odbl/1.0/). The Open Database License (ODbL) is a license agreement intended to allow users to freely share, modify, and use this Dataset while maintaining this same freedom for others, provided that the original source and author(s) are credited. Ben Walker, Michael H. Thurston, Guadalupe Bribiesca-Contreras, Tammy Horton
